# Case Report: *Nocardia farcinica* infectious arthritis and myositis in an immunocompromised host: diagnostic and management challenges

**DOI:** 10.3389/fmed.2026.1762442

**Published:** 2026-01-21

**Authors:** Jiacheng Liu, Yuanyuan Chen, Xiaowen Sheng, Yan Gao

**Affiliations:** Department of Infectious Diseases, Peking University Hepatology Institute, Peking University People’s Hospital, Beijing, China

**Keywords:** *Nocardia farcinica*, infectious arthritis, infectious myositis, muscle abscess, next-generation sequencing, drug sensitivity testing

## Abstract

**Background:**

*Nocardia farcinica* is a rare opportunistic pathogen predominantly affecting immunocompromised hosts. Infectious arthritis, cutaneous and deep soft tissue infections caused by this organism often present with nonspecific clinical manifestations. Additionally, due to its slow-growing and oligotrophic nature, both cultivation and identification pose considerable challenges, thereby complicating clinical diagnosis and management.

**Case presentation:**

This case report described an elderly female patient presenting with right shoulder redness, swelling, and pain. Her history included diabetes, local corticosteroid injections, and newly identified humoral immunodeficiency (hypogammaglobulinemia with low B-cell count). Imaging revealed infectious arthritis of the right shoulder, accompanied by infectious myositis and an intermuscular abscess in the right upper arm. *Nocardia farcinica* was confirmed by metagenomic next-generation sequencing (mNGS) and culture of aspirated fluid. Initial therapy with trimethoprim-sulfamethoxazole (TMP-SMX) and ceftriaxone was limited by renal impairment and gastrointestinal intolerance, and susceptibility testing indicated TMP-SMX resistance. Treatment was switched to linezolid. Due to inadequate clinical response, multiple surgical debridements were performed. Subsequently, therapy was changed to oral minocycline because of linezolid-induced bone marrow suppression and intolerance. At discharge, the maintenance regimen consisted of moxifloxacin combined with minocycline.

**Conclusion:**

This case highlights the importance of considering low-virulence pathogens such as *Nocardia* in immunocompromised patients with atypical infections that respond poorly to initial empiric therapy. Pathogen identification, aided by tools like mNGS for rapid detection, is essential. When classic regimens are limited by adverse effects, susceptibility-guided alternative therapies can be effective. For localized infections refractory to medical management, multidisciplinary surgical intervention remains a critical component of care.

## Introduction

*Nocardiosis* is an opportunistic infection caused by aerobic actinomycetes. It is uncommon in immunocompetent individuals but frequently occurs in hosts with impaired cellular or humoral immunity, such as organ transplant recipients, patients on long-term corticosteroid therapy, individuals with HIV infection, and diabetics. *Nocardia species* are ubiquitous in soil, water, air, and decomposing organic matter, primarily entering the human body via inhalation or through skin breaches, leading to suppurative infections. Clinical manifestations are diverse and nonspecific, often resulting in delayed diagnosis. The lungs are the most commonly affected organ, followed by the skin and central nervous system (1, 2).

Among *Nocardia species*, *Nocardia farcinica* warrants particular clinical attention due to its heightened invasiveness and distinct antibiotic resistance profile. This strain is associated with a greater propensity for disseminated infection, while primary infectious arthritis, myositis, or intramuscular abscess remains relatively uncommon. Its frequent resistance to third-generation cephalosporins further complicates empirical therapeutic decisions. Although TMP-SMX serves as the first-line treatment for nocardiosis, its clinical utility is often limited by adverse effects, including renal impairment, electrolyte disturbances, and gastrointestinal intolerance, which may preclude administration at optimal doses. Alternative agents such as linezolid and amikacin, which typically demonstrate good *in vitro* susceptibility, represent important therapeutic options (3–6). Consequently, when standard regimens are not feasible, identifying effective alternatives guided by susceptibility testing becomes essential.

Furthermore, when primary *Nocardia* infection is localized to deep soft tissues with abscess formation, medical therapy alone may be insufficient. For such patients, a multidisciplinary assessment to determine the necessity of surgical debridement constitutes a critical step in controlling the infection and eradicating the focus (7–9).

This case report describes an elderly female with diabetes and newly diagnosed humoral immunodeficiency, who presented with a rare primary *Nocardia farcinica* arthritis and intermuscular abscess of the right shoulder. The case highlights the pivotal role of mNGS in rapidly identifying uncommon pathogens, underscores the importance of susceptibility-guided individualized treatment strategies, and demonstrates the value of multidisciplinary collaboration between medical and surgical specialties in managing complex soft tissue nocardial infections.

## Case presentation

A 73-year-old female patient was admitted with a one-year history of intermittent right shoulder pain that had worsened over the past month. Approximately 1 year prior, she developed right shoulder pain with limited range of motion without an identifiable cause. She received local corticosteroid injections at an outside hospital, which initially relieved her symptoms. Subsequently, the symptoms recurred following the fixation and later removal of a right wrist fracture. Two additional corticosteroid injections were administered, providing only temporary relief. One month before admission, the right shoulder pain and functional limitation markedly worsened, accompanied by bilateral knee pain and lower-limb edema. A local betamethasone injection was given elsewhere, resulting in transient symptom relief lasting only 1 day before recurrence, along with local tenderness and swelling. She remained afebrile throughout this period. Multiple sessions of physical therapy and symptomatic intravenous treatments yielded no significant improvement. Two weeks prior to admission, she presented to our outpatient clinic, where two successive aspirations of fluid from the right upper arm were performed. mNGS of the purulent fluid confirmed infection with *Nocardia farcinica*. Antimicrobial therapy with linezolid, ceftriaxone, and sulfamethoxazole showed limited clinical efficacy.

The patient had a medical history of hypertension for over 20 years, diabetes mellitus for over 5 years, and hyperlipidemia for over 5 years. Additionally, she had undergone multiple previous surgeries, including cholecystectomy, combined myomectomy and ovarian chocolate cystectomy with sterilization, and appendectomy. Regarding personal history, she had long-term exposure to pet animals and reported no history of smoking or alcohol consumption.

**Figure 1:**
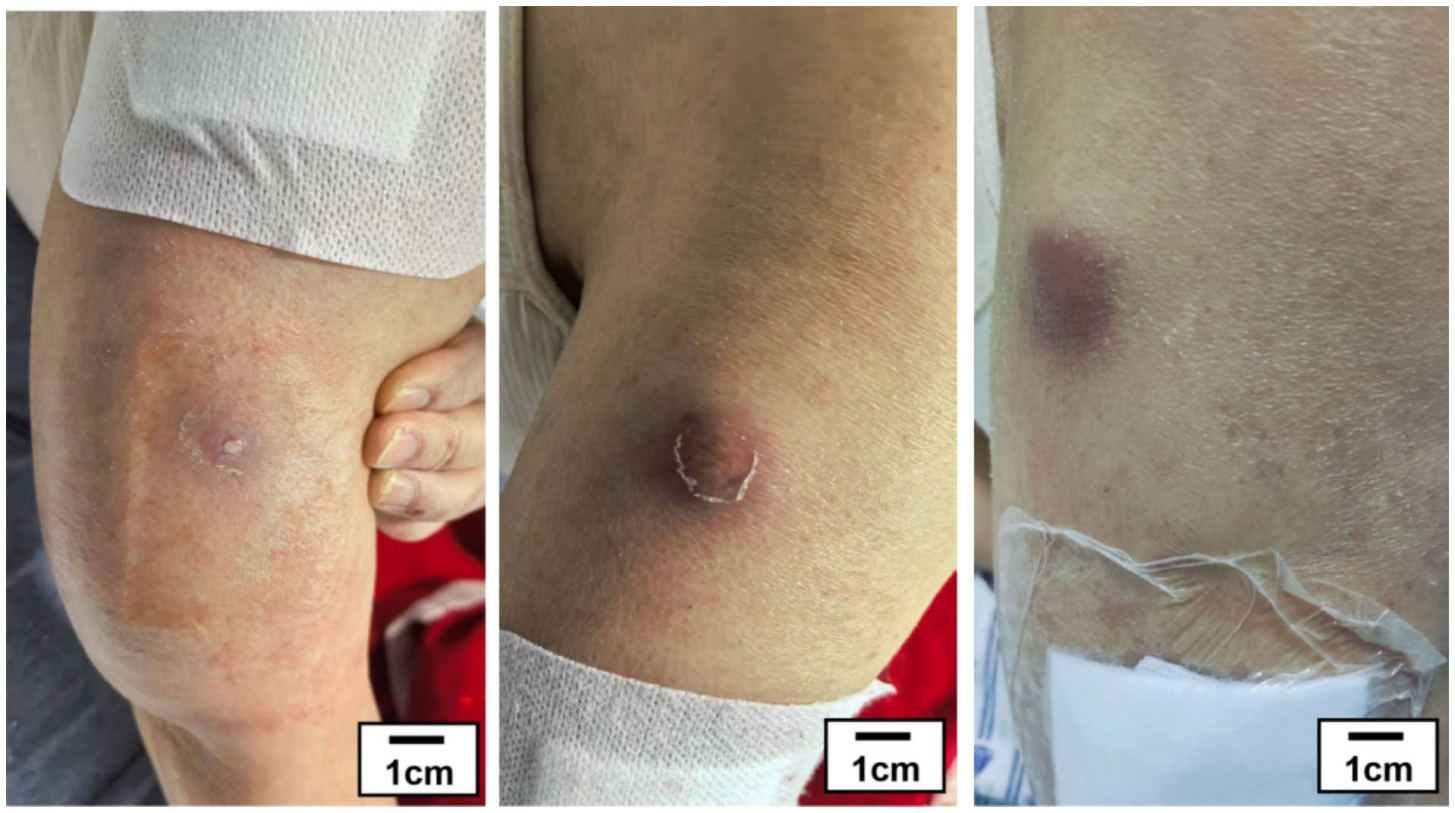
The preoperative appearance of the patient’s erythematous and swollen right forearm (scale bar provided).

Basic vital signs on admission were temperature 37.1 °C, pulse 97 beats/min, respiratory rate 18 breaths/min, and blood pressure 117/65 mmHg. Physical examination revealed subcutaneous masses over the right shoulder and the upper right elbow, with overlying skin showing redness, increased local temperature, and marked tenderness (Figure 1). Cardiopulmonary and abdominal examinations were unremarkable. Bilateral lower extremities exhibited pitting edema.

Laboratory tests from both external and our hospital indicated elevated inflammatory markers. Initial laboratory results from our hospital showed C-reactive protein (CRP) levels of 76.7 mg/L, erythrocyte sedimentation rate (ESR) levels of 98 mm/h, and ferritin at 393 ng/mL, while procalcitonin remained within the normal range. Complete blood count revealed microcytic hypochromic anemia. White blood cell (WBC), neutrophil, lymphocyte, and platelet counts were generally within normal limits. Serial laboratory results of the patient during the clinical course, including the complete blood count and inflammatory markers, are detailed in Table 1. Biochemical tests indicated hypoalbuminemia (albumin: from 35.22 to 24.2 g/L) and a transient deterioration in renal function (creatinine: from 73.3 to 103 μmol/L, later returning to 68 μmol/L). Liver function and electrolyte profiles were unremarkable, and coagulation parameters were essentially normal. Immunological workup demonstrated evidence of humoral immunodeficiency, with immunoglobulin G 6.37 g/L, immunoglobulin A 0.58 g/L, immunoglobulin M 0.39 g/L, and an absolute B-lymphocyte count of 65 cells/μL (reference range: 90–560 cells/μL). Additionally, the interferon-gamma release assay was positive, whereas the tuberculin skin test and Brucella antibody testing yielded negative results.

**Table 1:** Serial laboratory findings of the patient: complete blood count and inflammatory markers.

Date	Hospitalized duration	WBC (×10^9^/L)	NE (×10^9^/L)	HB (g/L)	PLT (×10^9^/L)	CRP (mg/L)	ESR (mm/h)	PCT (μg/L)	D–D (μg/L)
2025/10/20	–	5.99	4.10	100	331	76.7	98	0.089	553
2025/10/25	–	8.50	6.58	98	363	43.6	96	–	–
2025/10/29	Day 1	6.68	4.13	78	265	31.0	114	–	394
2025/11/02	Day 5	6.90	4.68	72	282	24.5	96	–	–
2025/11/07	Day 10	5.36	3.21	72	387	23.7	106	–	553
2025/11/13	Day 16	3.60	1.50	66	296	24.4	56	0.095	444
2025/11/18	Day 21	3.70	2.60	52	204	<0.5	48	0.100	317
2025/12/02	Day 35	2.10	0.50	99	171	32.4	7	0.068	403
2025/12/10	–	4.70	2.82	123	319	<0.5	–	–	467

WBC, white blood cell count; NE, neutrophil count; HB, hemoglobin; PLT, platelet count; CRP, C-reactive protein; ESR, erythrocyte sedimentation rate; PCT, procalcitonin; D–D, D-dimer; −, not tested.

**Figure 2:**
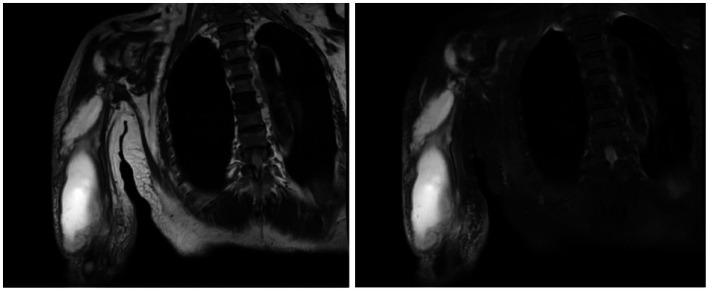
The preoperative MR enhancement scan (31st October 2025) of the humerus revealed multiple fluid collections, synovitis, and surrounding soft-tissue edema in the right shoulder and upper arm compartments, consistent with an infectious process.

Magnetic resonance imaging (MRI) of the right shoulder performed at an external hospital revealed changes suggestive of rotator cuff tendon and muscle injury, joint effusion, possible coracohumeral ligament injury, small cystic changes and medullary edema of the humeral head, and subacromial bursitis. Subsequent MRI of the right humerus and shoulder at our institution demonstrated multiple strip-like and patchy areas of high signal intensity within the right shoulder joint space, muscle compartments, and subcutaneous soft tissues of the right upper arm, accompanied by joint effusion. A posterior localized fluid collection communicating with the joint capsule was also observed, which appeared slightly hyperintense on diffusion-weighted imaging (DWI) and showed marked enhancement of the synovium and capsule wall on contrast-enhanced sequences, with mild-to-moderate enhancement of the surrounding soft-tissue strips (Figure 2). The imaging conclusion was multiple fluid collections, synovitis, and surrounding soft-tissue edema in the right shoulder and upper arm compartments, consistent with an infectious process. To evaluate for potential disseminated infection, further investigations were performed. An unenhanced chest CT scan revealed decreased bilateral pulmonary aeration and cardiomegaly, with no evidence of pulmonary abscess. Contrast-enhanced MRI of the head indicated a probable right frontal meningioma, scattered lacunar foci in the bilateral basal ganglia and centrum semiovale, mild white matter disease, and inflammation of the bilateral ethmoid and maxillary sinuses. No cerebral abscess was identified. Routine echocardiography revealed no evidence of valvular redundancy.

**Figure 3:**
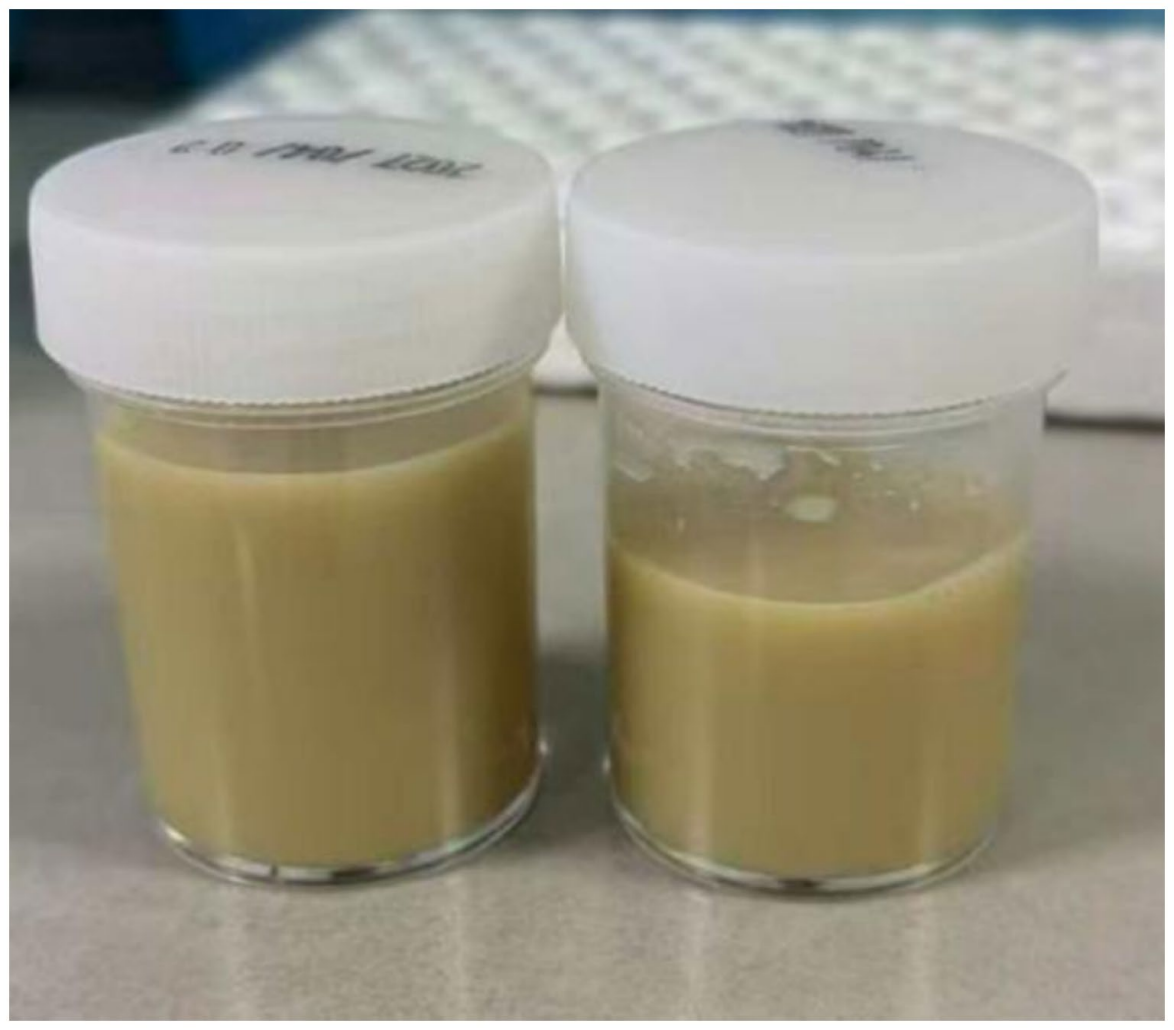
Fluid drained by puncture from an abscess in the right upper arm.

The definitive diagnosis was established through analysis of the yellowish-gray viscous pus (Figure 3) aspirated under ultrasound guidance from the right upper arm. mNGS of the pus detected *Nocardia farcinica* with 62,033 sequence reads (Table 2). However, cultures of specimens sent prior to admission remained negative, likely due to antecedent antibiotic exposure compromising bacterial viability, coupled with the fastidious growth requirements of *Nocardia* species. After hospitalization, repeat bacterial culture of the aspirated pus was performed. Through close communication with the microbiology laboratory and optimised incubation conditions, *Nocardia farcinica* was successfully cultured and antimicrobial susceptibility testing was completed. The results indicated susceptibility to ceftriaxone, imipenem, minocycline, and linezolid; intermediate susceptibility to doxycycline; and resistance to gentamicin, clarithromycin, and TMP-SMX (Table 3).

**Table 2:** The result of metagenomics next generation sequencing (mNGS).

Pathogen type	Species	Species-specific sequences	RPM ratio	Coverage	Relative abundance	Thresholds	Result
Bacteria	*Nocardia farcinica*	62,033	650.2	2,502,400 bp 39.16%	98.3%	≥1	Positive

**Table 3:** Results of the drug sensitivity test for *Nocardia farcinica.*

Smear result: Gram stain found G+ bacilli				
Cultivation results: *Nocardia farcinica*				
Bacterial colony count:++				
Antibacterial drug	KB/MIC folding point	Results	Experimental methods	Classification
Amoxicillin/Clavulanic acid	≥18 ≤ 13	27	KB	
Cefotaxime	≥26 ≤ 22	26	KB	
Ceftriaxone	≤8 ≥ 64	2	Etest	S (susceptible)
Cefepime	≥25 ≤ 18	25	KB	
Imipenem	≤4 ≥ 16	0.25	Etest	S (susceptible)
Gentamicin		6	KB	R(resistance)
Clarithromycin	≤2 ≥ 8	12	Etest	R(resistance)
Doxycycline	≤1 ≥ 8	1.5	Etest	I (intermediate)
Minocycline	≤1 ≥ 8	1	Etest	S (susceptible)
Ciprofloxacin		16	KB	
Trimethoprim/Sulfamethoxazole	≥16 ≤ 10	6	KB	R(resistance)
Linezolid	≤8	2	Etest	S (susceptible)

**Figure 4:**
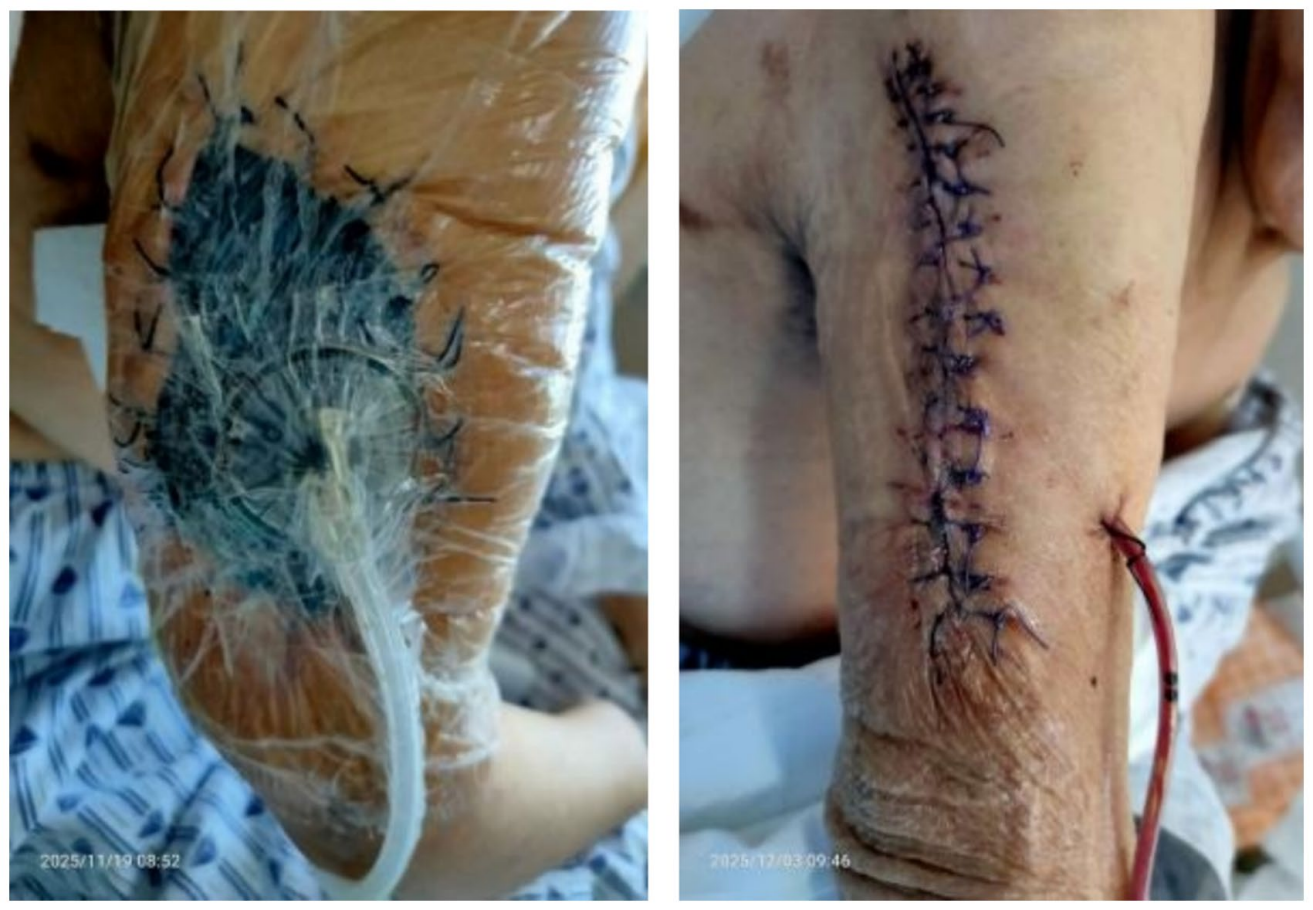
Postoperative appearance of the right forearm following surgical debridement and vacuum sealing drainage (VSD).

**Figure 5:**
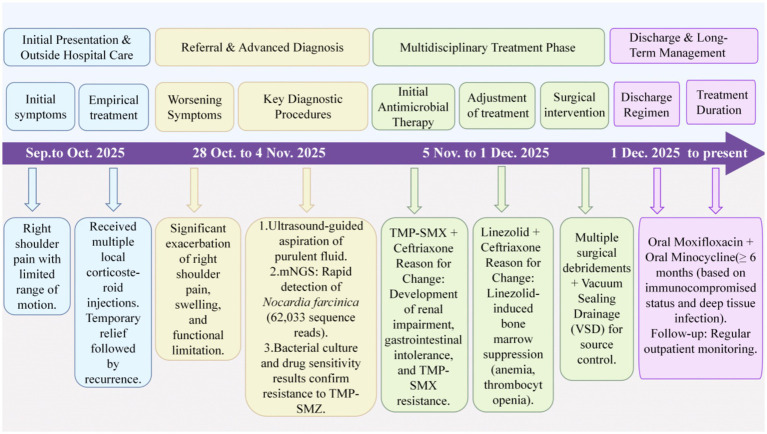
Flowchart of diagnosis and management for *Nocardia farcinica* infectious arthritis. The timeline outlines the key clinical events, diagnostic breakthroughs (notably the pivotal role of mNGS), antimicrobial therapy adjustments guided by susceptibility testing and adverse effects, and the essential multidisciplinary surgical intervention.

Following the detection of *Nocardia farcinica* by mNGS of the aspirated fluid, empirical antimicrobial therapy was initiated with TMP-SMX (800 mg every 8 h) combined with ceftriaxone (2 g once daily). Ceftriaxone was included to cover potential polymicrobial infection in this immunocompromised host, given its broad spectrum against gram-positive and gram-negative bacteria as well as anaerobes. However, during treatment, the patient developed renal function deterioration along with gastrointestinal symptoms such as nausea and vomiting, preventing the administration of TMP-SMX at an adequate dosage. Subsequently, based on antimicrobial susceptibility testing of the cultured isolate, the regimen was adjusted to linezolid (600 mg every 12 h) plus ceftriaxone (2 g once daily). Despite adequate antibiotic therapy, local swelling and purulent drainage from the right shoulder showed little improvement after over 2 weeks, indicating a suboptimal response to medical management alone. Given the persistent infection despite targeted antibiotics, radiological evidence of a deep-seated abscess, and the patient’s compromised immune status, surgical intervention was deemed necessary for source control. A trauma orthopedic team was therefore consulted to evaluate the need for surgical debridement. After thorough discussion with the patient, multiple surgical debridements were performed. Specifically, she underwent three consecutive procedures on November 10, November 17, and November 24, 2025, each involving debridement and suturing of the right upper arm combined with vacuum sealing drainage (VSD). A final debridement and suturing procedure was performed on December 1, 2025 (Figure 4). During this period, the patient developed progressive bone marrow suppression with declining hemoglobin and platelet counts, which was attributed to linezolid intolerance. Accordingly, guided by the antimicrobial susceptibility testing results, linezolid was replaced with oral minocycline (100 mg every 12 h, with a 200 mg loading dose). Following this adjustment, the patient’s blood counts gradually recovered, and inflammatory markers including WBC, CRP, ESR returned to normal. At discharge, the maintenance oral regimen consisted of moxifloxacin (0.4 g once daily) combined with minocycline (100 mg every 12 h). A total antimicrobial course of at least 6 months was planned, considering her immunocompromised status and the extent of deep tissue involvement. We will monitor patients closely on an outpatient basis. The diagnostic and therapeutic journey of this complex case is summarized in Figure 5.

## Discussion

*Nocardia farcinica*, an opportunistic pathogen, is prone to causing disseminated infections in immunocompromised individuals. It is most frequently observed in patients with impaired T-cell-mediated immunity, such as recipients of solid organ or hematopoietic stem cell transplants, those on long-term corticosteroid or other immunosuppressive therapy, individuals with malignancies (particularly hematological cancers), and people living with HIV. Diabetes mellitus represents another significant underlying condition, the associated microvascular complications and immune dysfunction are recognized as potential risk factors for nocardial infection (2, 10). Notably, clinical reports have indicated that *Nocardia farcinica* infection could also occur in immunocompetent adults, necessitating a high index of suspicion in this population. Diagnosis in such cases relies on a comprehensive assessment incorporating clinical history, imaging findings, and microbiological culture with extended incubation (11, 12). Furthermore, presenting symptoms such as generalized lymphadenopathy and fever of unknown origin may serve as important clinical clues, warranting prompt lymph node biopsy and mNGS testing to avoid missed diagnosis (13). In the present case, while the patient did not exhibit classic T-cell immunodeficiency, the presence of hypogammaglobulinemia and B-cell lymphopenia suggests that humoral immune dysfunction may also elevate the risk for nocardiosis. Additionally, her history of multiple local corticosteroid injections likely compromised the local skin barrier and immune microenvironment, potentially facilitating pathogen entry.

*Nocardia farcinica* infection primarily manifests as pneumonia, brain abscess, or cutaneous/soft tissue infection. However, diagnosis is frequently delayed or missed due to the nonspecific nature of its clinical symptoms and imaging findings. In immunocompromised hosts presenting with atypical pulmonary infection, such as subacute or chronic pneumonia showing nodules, cavities, or consolidations on imaging that is unresponsive to broad-spectrum antibiotics or with new neurological symptoms including headache, focal deficits, seizures, or altered mental status, a high clinical suspicion for nocardiosis, particularly the more invasive *Nocardia farcinica*, should be maintained (2, 12, 14–16). In immunocompromised patients presenting with limb abscesses that are unresponsive to conventional antibiotics and have a history of outdoor exposure, primary cutaneous nocardiosis should be considered (17, 18). Suppurative arthritis caused by *Nocardia farcinica* is rare, with shoulder joint involvement being particularly uncommon. Chronic joint pain accompanied by elevated inflammatory markers in immunocompromised individuals should raise suspicion for *Nocardia* infection (7, 19). Review of literature from 2016 to 2025 using PubMed for cases of infectious arthritis or myositis caused by *Nocardia farcinica* is detailed in Table 4 (7, 8, 19, 20). The patient in this case presented primarily with right shoulder pain and swelling. Imaging revealed an intermuscular abscess, a presentation easily mistaken for common bacterial infections or non-infectious conditions. Traditional etiological diagnosis relies on bacterial culture, but *Nocardia* grows slowly with a low positivity rate, often requiring 3–5 days or longer. In this case, mNGS applied to the aspirated pus rapidly identified *Nocardia farcinica* with a high sequence count (62,033), demonstrating clear superiority over conventional methods. Recent studies further support the significant advantage of mNGS in the non-invasive, rapid diagnosis of *Nocardia farcinica* infection. This is particularly valuable for immunocompromised hosts with complex infections, as it can quickly provide etiological evidence, guide early antibiotic therapy, and help reduce mortality (11, 14, 15, 21, 22).

**Table 4:** Review of literature from 2016 to 2025 using PubMed for cases of infectious arthritis or myositis caused by *Nocardia farcinica*.

Author	Study year	Age	Sex	Location of infection	Host-immune status	Therapy	Reference
Ishiguro et al.	2017	82	Male	Empyema, septic arthritis	Immunocompromised (pneumoconiosi, Type 2 diabetes mellitus)	Chest tube drainage, knee joint drainage, antibiotic therapy (ampicillin/sulbacta → minocycline → imipenem/cilastatin → levofloxacin)	(20)
Acuner and Cömert	2021	37	Male	Forearm subcutaneous abscess with fistula, multiple intramuscular abscesses	Immunocompetent	Surgical debridement and drainage, antibiotic therapy (TMP-SMX, amikacin, imipenem)	(8)
Thakur et al.	2023	78	Female	Right shoulder septic arthritis, periscapular abscess, lung	Immunocompromised (long-term prednisone and mycophenolate)	Surgical debridement, combination antibiotics (meropenem and TMP-SMX → amoxicillin-clavulanate)	(7)
Kessler et al.	2024	74	Male	Left knee septic arthritis, lung	Immunocompromised (mycophenolate mofetil, prednisone, and rituximab)	Arthrocentesis and surgical debridement, combination antibiotics (ancomycin and cefepime → linezolid)	(19)

Studies have indicated that among *Nocardia* species clinically isolated in China, TMP-SMX exhibited a low overall resistance rate and remained the first-line empirical therapy. Linezolid and amikacin demonstrated high susceptibility and served as important alternative or combination agents. In contrast, higher resistance rates have observed for ceftriaxone, clarithromycin, and tobramycin, necessitating regimen adjustments based on definitive species identification and susceptibility testing (3–5). In the present case, however, the isolate was resistant to TMP-SMX, and its use was further limited by the development of renal impairment and gastrointestinal intolerance. Recent research suggested that the sul1 gene was significantly associated with high-level TMP-SMX resistance in *Nocardia farcinica*, indicating a potential horizontal transfer mechanism for this resistance trait (23). Based on the susceptibility profile, therapy was switched to linezolid. With its excellent oral bioavailability, tissue penetration, and anti-nocardial activity, linezolid is a key alternative for patients intolerant or resistant to TMP-SMX, maintaining high *in vitro* susceptibility against *Nocardia* spp., including *Nocardia farcinica*. Nonetheless, its long-term use is constrained by significant adverse effects such as bone marrow suppression and neuropathy, requiring close hematological monitoring (24). Contezolid, a novel oxazolidinone, shows lower risks of myelosuppression and neuropathy compared to linezolid and demonstrates promising in vitro activity against clinical Nocardia isolates. It represents a potential new option, particularly for long-term management in patients intolerant to linezolid (25, 26).For complex or disseminated nocardiosis, combination therapy (e.g., with carbapenems, aminoglycosides, or fluoroquinolones) is often necessary and should be guided by susceptibility results (4, 27). Further studies have revealed that *Nocardia farcinica* exhibited the highest susceptibility to doripenem, followed by imipenem, with lower susceptibility to meropenem. Sitafloxacin and moxifloxacin showed significantly better susceptibility than ciprofloxacin, positioning them as viable alternative choices (28). Clinical reports have also suggested that a combination of moxifloxacin and high-dose minocycline could serve as an effective long-term maintenance regimen for severely immunocompromised patients with nocardiosis who were allergic to TMP-SMX, provided the choice was informed by susceptibility and pharmacokinetic principles (29). In our patient, linezolid was later discontinued due to bone marrow suppression manifesting as progressive anemia and thrombocytopenia. Treatment was successfully transitioned to oral minocycline based on the susceptibility results. Additionally, omadacycline has shown *in vitro* activity against most *Nocardia* species, with MIC values comparable to minocycline and tigecycline. Its oral formulation and favorable safety profile make it a potential alternative for nocardiosis, especially in patients intolerant to TMP-SMX or linezolid, though clinical trials are needed to confirm its efficacy (30).

Despite treatment with susceptible antibiotics, the patient’s local abscess persisted, indicating that medical therapy alone was often insufficient to achieve complete infection control once a deep-seated abscess has formed. Surgical debridement plays a key role in successful management by facilitating drainage, reducing the bacterial burden, and removing necrotic tissue. Literature highlights that immunocompromised status and diabetes are significant predisposing factors for *Nocardia farcinica* infection. For pyogenic arthritis or refractory subcutaneous abscesses caused by this pathogen, the combination of surgical debridement and susceptibility-guided antimicrobial therapy represents a standard, successful management approach. However, attention must be paid to susceptibility variations and contraindications related to underlying conditions, for example, avoiding macrolides in patients with myasthenia gravis. Long-term management typically requires repeated debridement when necessary, extended courses of sensitive antibiotics (often ≥6 months), and adequate psychosocial support (7–9). In this case, the infection was ultimately controlled through surgical debridement performed by the trauma orthopedic team in conjunction with tailored medical therapy. This outcome underscored the importance of multidisciplinary collaboration among infectious disease, radiology, and surgical specialties in managing complex nocardial infections. Following discharge, the patient was maintained on oral moxifloxacin (0.4 g once daily) plus minocycline (100 mg every 12 h) with regular outpatient follow-up.

Compared with previously reported cases of *Nocardia farcinica* infection, the present case demonstrates the following distinctive features. First, the infection site involved the shoulder joint and forearm muscle groups and interosseous spaces, rather than the more commonly affected lungs or central nervous system. Second, the host’s immune background consisted of combined humoral immunodeficiency and diabetes mellitus, rather than a single risk factor. Third, the treatment course evolved from a standard regimen to a susceptibility-guided individualized approach—prompted by drug resistance and side effects and ultimately incorporated surgical debridement. These characteristics broaden the clinical spectrum and inform management strategies for *Nocardia farcinica* infection.

## Conclusion

In immunocompromised hosts with atypical infections, obtaining a definitive microbiological diagnosis is crucial, with consideration given to low-virulence pathogens such as *Nocardia*. mNGS serves as a powerful tool for the rapid identification of such rare pathogens, although its results should ideally be correlated with culture and susceptibility testing. When first-line regimens cannot be administered due to adverse effects, susceptibility-guided alternative therapies represent effective options. For pyogenic infections that respond poorly to medical management, surgical debridement performed within a multidisciplinary framework, involving microbiology, radiology, surgery, and infectious disease specialties, constitutes a critical component of successful management.

## Data Availability

The original contributions presented in the study are included in the article/supplementary material, further inquiries can be directed to the corresponding author.
